# Health worker and patient views on implementation of smoking cessation in routine tuberculosis care

**DOI:** 10.1038/s41533-019-0146-6

**Published:** 2019-09-03

**Authors:** Melanie Boeckmann, Sahil Warsi, Maryam Noor, Omara Dogar, Esha Haowa Mustagfira, Fariza Firoze, Raana Zahid, Anne Readshaw, Kamran Siddiqi, Daniel Kotz, the TB & Tobacco Consortium, Ada Keding, Rhian Gabe, Anna Marshall, Steve Parrott, Shilpi Swami, Amina Khan, Sonia Raja, Salman Sohail, Rumana Huque, Deepa Barua, Samina Huque, Iashrat Jahan, Razia Fatima, Ejaz Qadeer, Aziz Sheikh, Helen Elsey, Jiban Karki, Eva Králíková, Iveta Nohavova, Kamila Zvolska, Alexandra Pankova, Sushil Baral, Shophika Regmi, Prabin Shrestha, Sudeepa Khanal, Basant Joshi

**Affiliations:** 1 Institute of General Practice, Addiction Research and Clinical Epidemiology Unit, Medical Faculty of the Heinrich-Heine-University, Werdener Str. 4, 40227 Duesseldorf, Germany; 2 Department of Health Sciences, University of York, Seebohm Rowntree Building, Heslington, York, YO10 5DD UK; 3 Department of Environment and Health, School of Public Health, Bielefeld University, Universitätsstr. 25, 33615 Bielefeld, Germany; 4 Independent Researcher, London, UK; 5 The Initiative, Orange Grove Farm, Main Korung Road, Banigala, Islamabad, 44000 Pakistan; 6 Plan International, Dhaka, Bangladesh; 7 ARK Foundation, Suite C-3 & C-4, House #06, Road #109, Gulshan-2, Dhaka, 1212 Bangladesh; 8 Usher Institute of Population Health Sciences and Informatics, Old Medical School, University of Edinburgh, Teviot Place, Edinburgh, EH8 9AG UK; 9 Department of Family Medicine, CAPHRI School for Public Health and Primary Care, Maastricht University, PO Box 616, 6200 MD Maastricht, The Netherlands; 10 NTP Pakistan, Islamabad, Pakistan; 11 University of Leeds, Leeds, UK; 12 VFN v. Praze, Prague, Czech Republic; 13 HERD International, Kathmandu, Nepal

**Keywords:** Respiratory tract diseases, Patient education, Health services

## Abstract

Smoking worsens tuberculosis (TB) outcomes. Persons with TB who smoke can benefit from smoking cessation. We report findings of a multi-country qualitative process evaluation assessing barriers and facilitators to implementation of smoking cessation behaviour support in TB clinics in Bangladesh and Pakistan. We conducted semi-structured qualitative interviews at five case study clinics with 35 patients and 8 health workers over a period of 11 months (2017–2018) at different time points during the intervention implementation phase. Interviews were conducted by trained researchers in the native languages, audio-recorded, transcribed into English and analysed using a combined deductive–inductive approach guided by the Consolidated Framework for Implementation Research and Theoretical Domains Framework. All patients report willingness to quit smoking and recent quit attempts. Individuals’ main motivations to quit are their health and the need to financially provide for a family. Behavioural regulation such as avoiding exposure to cigarettes and social influences from friends, family and colleagues are main themes of the interviews. Most male patients do not feel shy admitting to smoking, for the sole female patient interviewee stigma was an issue. Health workers report structural characteristics such as high workload and limited time per patient as primary barriers to offering behavioural support. Self-efficacy to discuss tobacco use with women varies by health worker. Systemic barriers to implementation such as staff workload and socio-cultural barriers to cessation like gender relations, stigma or social influences should be dealt with creatively to optimize the behaviour support for sustainability and scale-up.

## Introduction

Tuberculosis (TB) is a major global burden of disease, with 10 million people developing TB disease and an estimated 1.3 million deaths from TB in human immunodeficiency virus-negative persons in 2017.^1^ Of these cases, 87% occurred in high TB burden countries in Asia and Africa, with Pakistan and Bangladesh among the 8 countries with the highest numbers of cases.^1^ Pakistan accounts for 61% of the TB burden in the World Health Organization Eastern Mediterranean Region with 510,000 new TB cases yearly.^2^ In 2017, >360,000 new TB cases occurred in Bangladesh.^1^ TB is linked to tobacco smoking, and an estimated 15–20% of all pulmonary TB cases are associated with tobacco use.^3^ Tobacco smoking increases risks of a TB infection and worsens its prognosis through increased severity of symptoms, such as cavity lesions, continued increased likelihood of positive sputum and culture, decreased treatment adherence and increased risk of death.^3–5^

In Pakistan, tobacco use prevalence is estimated at 32% for men and 6% for women.^6^ In Bangladesh, male smoking prevalence remains at >30%, whereas women are perceived to rarely smoke yet do use smokeless tobacco products.^7^ It is projected that up to 18 million excess TB cases and 40 million excess deaths from TB could occur between 2010 and 2050 should smoking rates continue along their current trajectory.^8^ Persons with TB can therefore benefit from tobacco cessation support, especially considering that many immunological abnormalities in TB patients induced by tobacco smoking might reverse within 6 weeks of quitting.^9^ The TB diagnosis is a “teachable moment”^10^ to motivate people to re-assess their smoking behaviour, but standardized tobacco cessation is not yet widely offered in routine TB care in all high-burden TB countries. “TB & Tobacco”, a European Union-funded consortium, used an effectiveness-implementation hybrid design to study precisely this teachable moment by implementing a behavioural support intervention within TB care in Bangladesh and Pakistan. This occurs alongside a large randomized controlled trial to assess the effectiveness of cytisine (active vs. placebo).^11^

A brief behaviour support intervention was developed and tested with patients and health workers, consisting of a flip book, a leaflet and a poster.^12^ Materials can be downloaded for free on the project website https://tbandtobacco.org/. The key messages relate to managing TB and to quitting tobacco use, based on behaviour change techniques (BCTs) found effective in tobacco cessation interventions.^13,14^ Health workers received training in delivering the 15–20 min long intervention, focussing on tobacco use facts and on soft skills for communication and rapport-building with patients.^12^ At these trainings, health workers were also asked if they used tobacco themselves as part of a COM-B^15^ assessment pre- and post-training. The vast majority of health workers said they did not smoke (100% in Pakistan and 95% in Bangladesh).

Considering the high potential health gains for TB patients if offered smoking cessation, it is worth exploring the way our intervention was integrated into routine care and how health workers and patients perceived its acceptability, effectiveness and added value. What were the implementation processes at the clinic level in Pakistan and Bangladesh, and which barriers and facilitators to implementation on the ground might need addressing before country-wide scale-up? To answer these research questions, we conducted a process evaluation at three TB clinics in Pakistan and two TB clinics in Bangladesh. This paper reports findings on patient and health worker views regarding mechanisms of interactions between health workers, patients and the intervention and facilitators and barriers to successful integration of the intervention into routine TB care.

## Results

### Selected study sites

Out of 17 trial sites in Bangladesh and 14 trial sites in Pakistan, a total of 5 clinics were included as study sites. These included two case study sites per country where data were collected from patients and health workers and an additional fifth site in Pakistan where only health worker interviews were conducted. This site had originally been intended as a full case study site, but following low patient recruitment into the trial as many patients here had diagnoses of extra-pulmonary TB and were thus ineligible for recruitment, the site was closed and the case study shifted to another site to allow for full data collection including patient interviews. Characteristics of these case study clinics are described in Table 1.

**Table 1:** Case study site descriptions

Site	Country	Location	Type	Patient volume	Characteristics
Site 1	Pakistan	Urban	TB and chest diseases hospital	Very high	Only group counselling fit in with their patient flow. No option for private counselling
Site 2	Pakistan	Rural	TB Sanatorium	High	TB health workers already trained in behaviour support through other programmes and familiar with concepts. They were very receptive and active in the planning phases and trainings. The counselling room lacks privacy for counselling
Site 3	Pakistan	Urban	Tertiary care facility and teaching hospital	High	Very little time per patient. TB corner is separate with a combined waiting area with other departments and a private counselling room but very little space in the room for materials, records, etc. Hospital administration seemed less invested. This location was dropped and only health worker interviews were conducted at this site
Site 4	Bangladesh	Urban	NGO-run primary care clinic	Medium	Health workers had training on behavioural support through other projects. Engagement of management, in-charges and health workers in behaviour support development process, delivery and training
Site 5	Bangladesh	Peri-urban	Tertiary care facility	Very high	Very little time per patient, no private counselling area. TB counselling shared between staff

Only outpatient facilities were included in this study

*NGO* non-governmental organization, *TB* tuberculosis

### Provision of behaviour support: health worker views

We interviewed six health workers in Pakistan and two in Bangladesh, three women and five men (sample is described in Table 2). They ranged in age from 23 to 60 years and had been working with TB patients between 1 and 20 years; all were responsible for providing the behaviour support sessions. Their feedback on the intervention was generally positive, and they saw a need to offer smoking cessation support to their TB patients. Findings are reported by Consolidated Framework for Implementation Research (CFIR) construct for the health workers. Presented are those constructs that appeared frequently in the interview coding or that corresponded closely to our core research questions on how health workers interacted with the intervention and with their patients. Respondent DF4 was stationed at the clinic that was dropped at a case study site due to low patient enrolment into the trial, but behaviour support was implemented at this site and interviews with this health worker conducted.

**Table 2:** Health worker sample characteristics

Health worker	Age	Gender	Education	Time worked with TB patients	Country	Site
DF1	31	Male	M.A. in Education	10 years	Pakistan	Site 1
DF2	45	Male	Intermediate	20 years	Pakistan	Site 1
DF3	23	Female	B.A.	11 months	Pakistan	Site 1
DF4	29	Female	M.A. in Sociology	5 years	Pakistan	Site 3
DF5	40	Male	Intermediate	19 years	Pakistan	Site 2
DF6	29	Male	B.A. in Library and Information Sciences	9 years	Pakistan	Site 2
DF7	Late 50s	Male	Tuberculosis and leprosy control assistant degree	5 years at this site	Bangladesh	Site 5
DF8	52	Female	Paramedic	4 years at this site	Bangladesh	Site 4

*TB* tuberculosis

### CFIR construct: Patient Needs and Resources

Health workers’ responses focussed frequently on patients’ needs and resources and whether the materials were useful to patients:

*“Like I told you before flipbook is a better tool because when we are counselling a patient, what we say is not that effective but what we show with the help of pictures, for example when we tell them that if you smoke along with TB, your recovery will be slow and like that. Then the patient gets involved in pictures and listens carefully and pays more attention during counselling.”*

DF4, f, 29, Pakistan

Health workers adapted their delivery as needed specifically for patients with less formal education or who were unable to read and write:

*“[…] I altered the chart a bit; I consider that matter - whether the patients would be able to pay that much attention or not; so I have removed some part of the material; as well as marked some important part of it.”*

DF7, m, 60, Bangladesh

They were aware, however, that their efforts had limits and that the ultimate decision to quit was still the patients’ to make:

*“It is our effort that people should quit… we can only tell the patients that it is harmful…what else?”*

DF2, m, 45, Pakistan

### CFIR constructs: Self-Efficacy and Culture

The constructs of patient needs and resources overlapped with culture and self-efficacy in health worker statements: Asking women about smoking required that health workers felt capable of broaching this topic with the opposite gender, they expressed awareness of different educational and ethnic backgrounds among their patients and tailored their messaging accordingly, and adapted their approach to account for restraints stemming from patients’ fatigue or lack of time. For some health workers, approaching patients of the opposite sex regarding their smoking habits was more challenging than for others:

*“We cannot ask this directly. If we do, we will immediately receive a slap from her. (laughs) And she will say: ‘Don’t you feel ashamed of talking about cigarette with me? Are you joking? I am like your sister/mother.’ Such things start happening and one feels embarrassed in situations like these. But I directly rotate such situations and tell them: It is our responsibility and duty to ask you if you are having any problem other than that.”*

DF3, m, 31, Pakistan

If patients were found reluctant to disclose their smoking habits, health workers looked for some privacy to enquire about such questions:

*“Most patients tell us …teenagers who have hidden it from parents are reluctant to disclose in front of parents but we can see from their expression that they want to disclose but can’t so we provide them privacy and they tell us.”*

DF6, m, 29, Pakistan

In Bangladesh, health workers stressed the opportunity to save money spent on cigarettes for food for the family. Other cultural aspects included how to respectfully address patients, dealing with patients crowding the counselling room for fear of missing out on important information, and power relations between patients and health workers.

### CFIR constructs: Structural Characteristics and Costs

While the health workers were highly motivated and convinced of the relative advantages of the intervention, they had to deal with structural barriers to its implementation. These included high patient volume, infrastructure not conducive to counselling in private, high administrative workload and many patients not returning for follow-ups. The costs of implementation were therefore mainly health workers’ time and efforts, which caused problems of prioritization:

*“When I have to call the patient for flip book a lot of my time is taken up and my work starts piling up….while I am busy with flip book…number of waiting patients starts increasing….sometimes even to 14…some people are attracted and start listening…flip book is time consuming even though it is beneficial.”*

DF1, m, 31, Pakistan

The health workers requested support in dealing with the administrative tasks to free them up for counselling and to consider further reducing the number of pages and messages in the flip book:

*“So, the thing that we usually do in 5–10* *min takes us 20–25* *min. Let’s suppose we explain TB01 and TB02 card, what we can do is we can probably join 4-5 messages on TB02 card somehow.”*

DF5, m, 40, Pakistan

### Patient feedback on the behaviour support and intervention materials

A total of 35 patients participated in the interviews, with 4 from Pakistan and 3 from Bangladesh agreeing only to the first round of interviews (see Table 3 for description of the patient sample). Thirty-four of these interviewees were men. The participants’ ages ranged between 18 and 60 years, with a majority with low or modest incomes and some with limited formal education. Findings are presented by Theoretical Domains Framework (TDF) constructs that were frequently identified in the interviews or that answered the research question on patients’ smoking behaviour after the intervention. We observed similar responses to the questions on satisfaction with the intervention across the patient sample.

**Table 3:** Patient sample characteristics

Characteristics	Pakistan	Bangladesh	Total
Total	18	17	35
Gender			
Female	1	0	1
Male	17	17	34
Age, in years			
18–25	3	6	9
26–35	6	6	12
36–45	2	2	4
46–55	7	1	8
56–65	0	2	2
Occupation			
Unemployed	3	2	5
Manual labour	4	4	8
Driver/taxi driver	5	0	5
Businessman or employed^a^	3	11	14
Teacher/preacher/office worker	3	0	3

^a^Employed = any other job held as employee (not self-employed, none of the other categories listed here)

### TDF construct: Behavioural regulation and breaking habits

The participants frequently reported making quit attempts and changing their smoking behaviour; many linking their willingness to quit with their TB:

*“It was a nice explanation that health worker gave. The way he spoke to me, all the words matched with my heart and soul that I realized, it is better to give up. I felt pity for myself as I have become very feeble. My disease increased due to smoking, for that I also had plans to give up. Now, I have given up smoking and it is been a week since I totally got rid of smoking.”*

P30, m, 25 years old, Bangladesh

The potential effect on other family members in case the breadwinner remains sick or even dies was also a motivator to quit smoking:

*“I think it came from understanding the importance of staying alive. I am ill. And if I do not recover then how will my family function. Furthermore, I will not survive. So it was from that realization that I decided to stop. If anything happens to me then my children and family will suffer. I will not exist and they will suffer. This is mainly why I quit smoking.”*

P22, m, 40, Bangladesh

While support from the health workers was perceived to be an eye-opener to the importance of quitting, the majority of participants felt that their quit success relies on their willpower:

*“Smoking… it’s not good. What I’m doing is not legal, not right. I also know that it’s not good, but I did. […] It’s all about our heart, how we feel and act. It’s up to us if we want to quit or not. I quit smoking earlier as well, like during Ramadan I didn’t have cigarettes. I quit smoking for the sake of fasting, didn’t break a single fast! Kept the spirit up for one month; 30 long days of fasting, yet I started again. My weakness got me again. It’s all in the brain, nothing else. Just the brain got into evil spirit again.”*

P26, m, 40, Bangladesh

In Bangladesh, the chance to save money by quitting was also a common reason given for making quit attempts:

*“I stopped all by myself as it’s difficult to go to relatives, children, wife. They also have a future. It costs 200 to 150 taka to smoke. Instead, if I eat actual food like rice curry with my wife and kids, it’s far better.”*

P23, m, 25, Bangladesh

A few patients reported “cutting down to quit” as a preferred option rather than setting a quit date and following the “not a puff” rule. “Not a puff” means committing to quitting abruptly and to not smoking at all to avoid continued nicotine exposure:

*“Yes, I am trying. It is quite better than before. I am trying a lot*.

*I [Interviewer]:*

*What do you mean by saying ‘trying’?*

*P20:*

*What? Before if it was 5 now it is 1 or 2. That is how you can stop but it will need a bit of time. You cannot leave it at once.”*

P20, m, 18, Bangladesh

Dealing with cravings was an important topic of conversations. The majority adhered to professional advice on substituting smoking with another habit such as taking lozenges, asking friends and family for support or distracting oneself to deal with cravings and withdrawal symptoms. Others ranged from avoiding all social contact and staying indoors to physically breaking other people’s cigarettes when they smoked in front of the patient:

*“I:*

*When you had the craving for a cigarette, how were you able to resist that?*

*P32:*

*When I had cravings, I did not go near cigarettes. I did not even go near to any shop. I remained seated in my home all the time. I did not go outside at the time of craving; rather I remained in my home, continuously.”*

P32, m, 24, Bangladesh

*“I just keep on asking [my family member] to quit. What else do I have to feel? If he smokes around me and if I get hold of his cigarette, I just break it.”*

P9, m, 50, Pakistan

Overall, behavioural regulation was connected to beliefs in one’s capabilities, beliefs about consequences and social influences.

### TDF constructs: Social and Environmental influences

The three social groups mentioned in connection to smoking were family, friends and work colleagues. Social environments rather than physical environments mattered to patients’ narratives:

*“My mother was very upset with me due to my smoking habit…they all do try to keep me from smoking.”*

P13, m, 35, Pakistan

*“It was just yesterday that a friend of mine was offering me a paan. He said one paan will not make any difference. But I told him: ‘No, I have quit’. So, I did not have it.”*

P5, m, 34, Pakistan

### TDF construct: Beliefs about consequences

Offering cytisine together with behaviour support was seen as a successful approach by patients:

*“To get rid of this habit, this medicine helps you completely. You have to make a decision for yourself, of course, but the capsules that I got from RA [research assistant] help a lot in this.”*

P7, m, 52, Pakistan

Social groups strongly influenced behavioural regulation and person/environment interaction. Family support was the most frequently cited contributor for quit success besides willpower, and pressures from work colleagues and friends to keep smoking were barriers to cessation.

Beliefs about consequences were linked to quitting, failing to quit and to the consequences of receiving cytisine. Quitting smoking was identified to have multiple benefits for one’s own health and for one’s family:

*“I told them that they did this for my own good. The money I spent on smoking, I will buy milk for you with that instead. That way, my health will stay better. That was harming me. I understood that I am better after I quit smoking.”*

P23, m, 25, Bangladesh

*“I:*

*Why do you think that it was better to quit smoking?*

*P26:*

*I smoke now but when I gave up smoking, I gained weight. I have a child, I have to feed him/her, and my child can be affected though I told them that I do not smoke now.”*

P26, m, 40, Bangladesh

A large number of patients reported that they believed the medication helped them quit, even when it was uncertain whether they had received cytisine or placebo.

*“I:*

*Can you please elaborate on your experience of quitting smoking?*

*P28:*

*After consuming the drugs, a strange thing happened. When the smell of cigarettes entered my nose I felt like vomiting. I simply cannot tolerate the smell now. Not at all. I was surprised to see this change in me which happened after I quit smoking and had the drugs.”*

P28, m, 27, Bangladesh

### TDF construct: Emotion

Although the original TDF construct Emotions focusses on negative emotions, we decided to include positive feelings in its description for our project. Stigma and shame about being confronted about one’s smoking habit were also coded with this construct. While many patients in Bangladesh did not keep their smoking habits a secret, their TB diagnosis was often a delicate matter:

*“I:*

*That’s good. Did you talk about quitting tobacco with your family or friends after the session at the hospital?*

*P23:*

*No, I didn’t talk to them about this [TB]*.

*I:*

*Nobody knows?*

*P23:*

*No*.

*I:*

*Then what do they do?*

*P23:*

*If they knew, they will not be communicating with me, they’ll stay away from me. That’s why I didn’t tell them.”*

P23, m, 25, Bangladesh

Among Pakistani respondents, tobacco use was perceived as more illegitimate than among the Bangladeshi participants, although the majority of respondents expressed that they were not embarrassed about admitting to smoking:

*“No No No…it was very easy for me to talk about it. My parents, wife and five kids live in village. It’s not that they will write something about smoking in my file and they will beat me up. I live alone so I am not afraid of anyone. It is my decision.”*

P10, m, 30, Pakistan

As expected, the sole female interviewee in Pakistan expressed strong concern about societal and familial reaction to the discovery of her smoking habit:

*“If I smoke cigarettes and hookah, I do it keeping it hidden from my father because if he knows about it, he would beat me. It is not allowed in my family. So, when they were talking to me about quitting intoxications and all, were all surprising for me. They were saying that they would give me a treatment but I was feeling startled about it.”*

P15, f, 25, Pakistan

Emotions were also associated with giving up smoking, some very positive but some with a little regret:

*“I felt irritated and felt like something was missing, there was an emptiness all the time. There was a lack of something that I always used to have. Like this, something felt gone.”*

P28, m, 27, Bangladesh

While emotions were expressed as part of participants’ reactions to the intervention, they were not portrayed as a major barrier or facilitator to cessation.

### Emerging constructs: Perceptions of the intervention process and of the TB diagnosis and therapy process

Patients remembered the counselling sessions and expressed gratitude to be given advice and support. None of the patients had suggestions for changes to the intervention nor did they criticize what we perceived as shortcomings, such as lack of private rooms for counselling, infrastructure age and state, long wait times or being asked directly about smoking habits.

The advice given by health workers was appreciated:

*“Bhai [a polite way to address a male person in Urdu; meant to mean the health worker] told me many things, made me understand. Umm… he gave me medicines. […] He told me that smoking is bad, bad for health. It creates problems, for me, for the people around me, for everything. Mostly it makes me sick. It’s not good for health. And the money I spend on smoking is basically wastage of money. […] He made me understand that I can save this money for some other need or necessity. I may buy fruit and have it instead of cigarettes, or may be something nice for my kids, why waste it on smoking? These are the things he told me and made me understand.”*

P26, m, 40, Bangladesh

Narratives of the long process from first TB symptoms until diagnosis and referral to the health workers indicated stressors for patients that were not directly linked to the intervention.

### Emerging code: Religious beliefs

Expressions of faith are part of communicative rules in both Bangladesh and Pakistan. In some instances, beliefs in capabilities to quit were linked to support from God:

*“I:*

*That’s good, how do you feel about quitting smoking now?*

*P21:*

*Yes, a bit difficult but better now. Inshallah. If Allah wants I’ll continue like this. I am coping with all.”*

P21, m, 60, Bangladesh

Similarly, the expectation whether or not one can be cured of TB was linked to faith:

*“P23:*

*Everyone at home knows*.

*I:*

*Can you please explain a bit?*

*P23:*

*I informed them, told them about my disease, medicine, smoking, quitting. What else would they say? They are just crying. I told them what’s the point? Only Allah grants us the permission to live and die.”*

P23, m, 25, Bangladesh

### Cross-country differences in patient experiences

Faith was expressed more often in the Pakistan sample, particularly in relation to willpower to quit cigarettes and regarding beliefs about whether TB will be cured. In Bangladesh, patients more frequently listed money as a benefit of tobacco cessation, which is likely linked to the Bangladesh intervention materials that, other than the Pakistan materials, included money as one additional aspect. All male participants reported less stigma related to tobacco smoking than expected, although some discomfort upon being asked about their smoking was expressed in their responses. Health workers were partly concerned about asking patients directly if they smoke, which is a discrepancy to patients’ views.

## Discussion

The TB & Tobacco behaviour support intervention for tobacco cessation for TB patients was largely successfully implemented in routine TB care at four case studies in Bangladesh and Pakistan, while one case study site had to be dropped due to low recruitment.

This study provided insights into the realities of offering a smoking cessation behaviour support intervention in TB clinics in Bangladesh and Pakistan and, to our knowledge, is one of few qualitative studies reporting the process evaluation of a multi-country smoking cessation implementation study in patients with TB, collecting both patient and health worker perspectives at multiple time points. Based on constructs identified in the interview analyses, our findings suggest that the following aspects of the behaviour support intervention were perceived as useful by health workers and patients:

- Targeting the TB diagnosis as a teachable moment for tobacco cessation: Patients reported that learning about the impacts of smoking on TB progression was a motivating factor for quitting.
- Having picture-heavy materials available to illustrate messages: Health workers felt comfortable showing pictures to patients and assumed these would aid in understanding the main messages.
- Providing relatable reasons for cessation such as health, family, saving money to afford essentials: Patients reported that social situations with friends, family or at the workplace influenced their smoking behaviour.

These findings on the importance of images are in line with prior research on BCT-based cessation interventions in South Asia.^16–18^ Patients were not opposed to being approached about their smoking habit, although health workers expressed concerns that women might not admit to tobacco use and that asking about tobacco use was a sensitive issue.^12^ There is a need for more in-depth research on what role stigma really plays and whether gender remains a determinant for smoking even among younger generations of women in Bangladesh and Pakistan.^19–21^ We cannot be sure to what degree our anticipation of hesitancy in admitting use might have been part of our training and our interaction with the participants and hence shaped their responses more than actual concerns.

Interviewed patients predominantly stated they had made successful quit attempts 4–6 weeks after being counselled. Similarly to findings from high-income contexts, social environments played an important role in dealing with cravings and withdrawal symptoms,^22,23^ and a strong belief in willpower has also been described in tobacco cessation research in Western countries and in Oceania.^24–26^ The role of religious convictions in supporting cessation attempts was not a targeted aspect in the intervention or process evaluation, but other studies are investigating this phenomenon^27,28^ and it could be considered for future adaptations of the programme.

Structurally, health workers were highly motivated to support their patients to quit smoking, yet they were burdened with administrative tasks, shifts among colleagues, high patient volumes and lack of acknowledgement of their accomplishments at some clinics. While the intervention is designed to be integrated into counselling that already takes place, in reality work routines were not always conducive to such changes and caused additional time burdens. To address these challenges, support from National TB Programmes and clinic heads to reduce documentation burden and ensure regular refresher training seems to be essential, as has been shown elsewhere.^18^

Patients and health workers problematized the following aspects of the intervention process:

- Being willing to admit to smoking at all depends on the individual patient’s comfort level: Health workers felt a need to make sure there was privacy and trust before asking patients directly about their smoking habits. This was especially the case for male health workers asking female patients.
- Adding the counselling process to the diagnosis and TB counselling often requires more time than the health workers feel they have available: The duration of the intervention was discussed frequently.

Overall, the intervention appears to address a need at clinics and was welcomed by participants.

Throughout this qualitative process evaluation, different levels of power relations played a role: between researchers and respondents, patients and health workers, and health workers and their employers. Interviewers in Pakistan and Bangladesh as well as the data analysts were members of the TB & Tobacco research consortium and were invested in the success of the project. Their status as researchers and doctors likely also influenced how respondents reacted to them, particularly related to the gap in social status between the patients and the interviewers. Our interviewers were all female; almost all patients interviewed and the majority of health workers were male. This added another layer of power differentiation to the interview situation.

As this study was conducted as part of a pragmatic clinical trial in lower-resource settings, some limitations occurred during data collection and analysis. Several round two patient interviews were conducted by phone rather than face-to-face, in cases where the patients did not return to the clinics as expected. Patients were asked to be interviewed on the day they received their TB diagnosis. They were often tired, had already been at the clinic for a long time and were weak from their illness. As a result, the sampling approach had to be pragmatic as fewer patients were willing to talk to us. Gender bias in both countries also greatly reduced our opportunities to interview women. In addition, as interviews had to take place at the clinics to avoid further time burden or risk exposing patients’ illness to their social environments by interviewing them at their homes or work places, the interview situation was characterized by noisy surroundings, lack of complete privacy and potential interruptions. Interviewees may not have been completely open during these interviews if other patients or health workers were nearby. In Pakistan, patients were interviewed in rooms a bit further away from the health workers to ensure as much privacy as possible. In some instances, a male research assistant accompanied the female interviewer if the interviewee was male and the interview location a more isolated room. Patients were also interviewed while receiving TB treatment. It is possible that they worried about quality of their care, or felt indebted to the researchers in a way, and thus possibly gave socially desirable answers. While health workers were given the option to be interviewed in a different setting, they preferred their work places to save time and be in a familiar environment. Site number 3 had to be dropped as a case study site due to the trial requirements. While BS implementation occurred and interviews with the health worker at this site were conducted, we were unable to get patients’ viewpoints on the process at this clinic.

Owing to staff re-organization, some follow-up interviews with patients or health workers were conducted by a different interviewer, which could have influenced willingness to respond. We examined the possibility of social desirability by looking at both interviews taking place face-to-face at clinics and those conducted via phone to assess whether there are discernible differences in patient views on the intervention. We found neither large discrepancies between the two groups outside of the expected difference between individuals’ perceptions nor any patterns of satisfaction or dissatisfaction.

All interviews were conducted in the interviewees’ and interviewers’ native languages, then transcribed by bilingual speakers directly into English. While this enabled a multi-professional and multi-national analysis process, we cannot rule out translation errors or errors in ascribed meanings. To counter this problem, all analysis occurred either in a cross-country team or, as with the Bangladesh data set, country teams gave feedback on coded text segments. We chose a case study approach to conduct in-depth research at selected clinics to gain insights into practices on the ground and in the “real world”. This approach was a good fit for the research question of how the implementation of the behaviour support intervention was perceived. Different project work packages, such as a quantitative process evaluation component and the context evaluation, will help fill in potential gaps left by this interview study.^29^

These stated limitations give good insights into implementation challenges at the clinic level: issues of lack of privacy, noise levels, or patients not returning for follow-ups are equally relevant for the intervention implementation and scale-up. Our limitations thus highlight areas of particular concern for future iterations of the behaviour support programme. On the other hand, this study has multiple strengths. All research was conducted by multidisciplinary teams from Europe and from Pakistan and Bangladesh who were equal members of the research consortium. Local expertise was essential to the success of the interview study. Having interviewers from the project team who had previous working relationships with the clinics provided a level of familiarity and trust that made it possible for the health workers to open up about their experiences. We collected qualitative data from different perspectives and at different time points, which is unusual for trial process evaluations and adds value. Having longitudinal interview data from both patients and health workers allowed us to better understand how the intervention was integrated into routine care in the medium term and how patients interacted with the knowledge and skills provided during the counselling after they left the clinic. As the interviewers not only visited the clinics for the interviews but also frequently for other tasks related to the trial, they were able to gain further insights into mechanisms at the clinics and to become a more accepted presence at sites. Interviewers were culturally and socially familiar with contexts at sites and spoke participants’ languages. Interviews were coded by two coders (Pakistan) or by one coder with coding discussions with the larger team (Bangladesh) to include multiple perspectives in the interpretation of findings.

We triangulated the interview data with health worker notes on non-participation, field notes accounting direct observation of researchers from site visits and with feedback from other project researchers who were not part of the qualitative assessment yet were deeply involved in other aspects of the study. This added validity to our data and provided a more comprehensive picture of the implementation status at sites.

Most frequently raised points include that intervention materials appeal to patients and health workers and the TB diagnosis is a teachable moment that can be harnessed for cessation support. However, high workloads for health workers in the TB clinics, stigmata regarding women who smoke and social influences on smoking behaviour remain challenges that need to be dealt with creatively for sustainable scale-up and long-term implementation.

## Methods

Details of the behaviour support intervention, the cytisine trial and the process evaluation are published in the process evaluation and the trial protocols.^11,29^ In short, regarding the behaviour support intervention, we aimed to systematically assess the implementation process at clinic level and to discover mechanisms of interaction between participants and the intervention by observing and interviewing patients and health workers. For the qualitative process evaluation components, we chose a multi-site case study design^30,31^ to gain an understanding of the multi-faceted and complex real-life contexts at the clinic level. In deviation from the protocol, the qualitative findings are reported here separately from the quantitative findings, as the trial was delayed yet behaviour support implementation had started in 2017.

Study site selection reflected key dimensions of difference among facilities in each country. We included clinics from varying healthcare levels (primary, secondary or tertiary), different staff size, and from both urban, peri-urban and rural settings. Selection also accounted for site conditions regarding work flows and willingness of clinic heads and staff to participate in the study. We only included sites that had medium-to-high patient flow to ensure adequate interview participant sample sizes among patients.

### Data collection

To assess intervention implementation, semi-structured interviews (SSIs) were conducted with health workers from five case study sites and with patients from four sites (see section on “Interviewee sampling” below). The interview guides (see Supplementary Files 1 and 2) were developed during the first phase of the study in a larger team consisting of researchers from the UK, Czech Republic, Germany, Pakistan, Bangladesh and Nepal. Guides were written in English, translated into Urdu and Bangla by native speakers and translations checked against original English versions. Interview guides covered contextual, implementation and interaction aspects (Table 4). The Pakistani interviewers (M.N., R.Z.) wrote memos on the interview situations that were used as contextual data to support the analyses. One of the four interviewers (M.N.) was also involved in data analysis.

**Table 4:** Interview topics

Topics included in health worker interview guides	Topics included in patient interview guides
•Perceptions on intervention materials •Perceptions on delivering the behaviour support intervention •Further training and support needs •Interactions with patients •Recommendations for future programme adaptations	•Patients’ experience of behaviour support received •Perceptions of intervention materials •Family and friends’ tobacco habits •Tobacco rules at patients’ work places •TB medication and tobacco cessation regimens burden •Social support for quitting tobacco and potential stigma •Tobacco availability near the clinic, patients’ houses and work place •Recommendations for future programme iterations

### Study participants: sampling for interviews

#### Health worker sampling approach

At each case study site, all health workers predominantly in charge of providing directly observed treatment short course for TB were trained in March 2017 to deliver the behaviour support session as part of their routine counselling. These health workers were invited to be interviewed at three different time points over a period of 11 months to examine the development of intervention implementation. We opted for a longer data collection phase to capture health workers’ views early in the implementation process, in the middle and towards the end of the study. This allowed us to assess whether attitudes and practices changed over the project duration, after health workers became more familiar with the counselling process and the project was integrated into clinic routines. In Pakistan, five health workers (pseudonyms: DF1, DF2, DF4, DF5, DF6) from three sites were interviewed at baseline (July to September 2017), four (DFs 1, 2, 4, 5) at the second time point (December 2017) and four (DFs 1, 3, 4, 5) at round 3 (April to May 2018) for a total of six different interviewees. None of the trained health workers refused to be interviewed. The sample size between interview rounds differed owing to health worker re-assignments. One preliminary case study (Site 3) was removed from the trial due to low patient recruitment, necessitating cessation of patient interviews at the site. A new case study site (Site 2) was initiated, subsequently leading to new interview partners among the health workers. In Bangladesh, the same two health workers from two sites were interviewed three times (baseline: October 2017; second round: January 2018; third round: May 2018). Interviews with health workers were conducted at their work place to allow participation without asking them to take unpaid time off.

#### Patient sampling approach

Patients enrolled in TB care, who had agreed to participate in our study at the case study sites, were purposefully sampled. They were approached face-to-face after leaving the consultation with the TB health worker and were invited to participate in two SSIs. Patients approached to be interviewed had to have received the behaviour support counselling, be enrolled in the trial and be able to give consent. Those who refused to participate did so mainly on grounds of lack of time. The purposeful sampling approach was pragmatic and aimed to capture a range of voices to identify how different people interacted with the intervention and to hear their input on possible intervention changes. We estimated that a sample of 5–10 interviews per case study site^32^ would be a realistic goal to hear perspectives from different age groups, occupational and educational backgrounds and smoking histories required for the narrow scope of the research question.^33,34^ Our sample size was determined using Malterud et al.’s concept of “information power”^34^ for qualitative research, which ensures information maximization for smaller study samples by thinking through five domains. In our research, four domains suggested a smaller sample size given (a) the narrowly defined aim to identify facility-level implementation barriers and enablers, (b) a specified sampling of participants to represent a cross-section across facilities and time points, (c) application of CFIR and TDF theoretical frameworks to data collection and analysis, and (d) incisive and in-depth dialogue between researchers and participants. The final domain of having a cross-case analysis strategy for interviews suggested widening the sample size to ensure a representative sample across the facilities and countries.

Owing to the gendered nature of smoking in Bangladesh and Pakistan,^7,35^ women rarely stated using tobacco and were not willing to be interviewed on this topic, with one exception in Pakistan. While a private interview situation was sought, at the sites a private room was rarely available, and some patients asked for spouses or family members to stay present during the interview.

The two interviewers in each country (M.N., R.Z., F.F., E.H.M.) were members of the core TB & Tobacco research team and were involved in several study work packages in addition to the process evaluation. All interviewers had previous research and academic experience, were trained in conducting SSIs and were fluent in their native languages and in English.

#### Consent procedures

Participants’ written consent was received prior to data collection. Consent forms were adaptable for illiterate patients as they allowed signature by thumbprints if needed. Selected patients who were no longer returning to the site for medication pick-up or were otherwise physically unavailable but who indicated willingness to participate in follow-up interview rounds were interviewed by phone. In these cases, verbal consent was taken over the phone and recorded, and written consent provided at the next trial follow-up appointment after the interview.

#### Ethics approval

Ethical approval for all aspects of the qualitative data collection was gained for all sites (University of Leeds (MREC15-063), Bangladesh Medical Research Council and Bangladesh Drug Administration (BMRC/NREC/2016-2019/1475), Pakistan Medical Research Council (4-87/16/NBC-200 Part-B/RDC/4/97)).

### Data analysis

#### Interview analyses

Interviews were audio-recorded, transcribed directly into English from Urdu or Bangla and analysed using a combined deductive and inductive approach based on the CFIR^36^ and the TDF.^37,38^ This tailored framework approach was used to capture organizational aspects of implementation as well as individual voices. CFIR has been used to analyse and evaluate complex interventions in lower-income settings^39^ and focusses on factors relevant to implementation success across five domains: intervention characteristics, outer setting, inner setting, characteristics of individuals, and process. As CFIR focusses strongly on implementation at an organizational level, TDF was used to capture patient voices as it includes constructs such as emotion that are missing from CFIR.

Each country data set was coded separately and then combined to explore cross-country similarities and differences. Among the authors, M.B. from Germany and M.N., who conducted the majority of interviews in Pakistan, analysed the Pakistan data set together with Esther Scholz, who was not involved in the study. The Bangladesh data set was coded by M.B., with discussion on selected coded sections with a multi-professional team of the TB & Tobacco consortium. This means that, in the Pakistan data set, all interview transcripts were coded by two researchers independently. In the Bangladesh data set, all transcripts were coded by one researcher and findings presented and discussed to a group within the research team.

Health worker and patient interviews were analysed by country, using the NVivo 11 Pro and MaxQDA 11 software, as separate sets. Health worker interviews were coded in two rounds, first employing only the CFIR framework, and then identifying and applying emerging constructs. Similarly, the first round of patient interviews only used TDF constructs, with emerging codes added and used in the second round. See Supplementary Files 3 and 4 for the codebooks. Summary memos were developed for each case study site containing the primary identified constructs with supporting quotations from health workers and patients.

Owing to patient fluctuation at the sites, shortage of time among patients and health workers and high illiteracy among patients, we were unable to return transcripts to participants for checking. We thus relied on feedback from the research teams to verify findings from transcripts. Members of the research teams spent significant time at the sites during the trial and implementation period and were presented with coded sections and findings during the analysis process.

### Reporting summary

Further information on research design is available in the Nature Research Reporting Summary linked to this article.

## Supplementary information

Supplementary Information.

Reporting Summary

## Data Availability

Full data sets are not publicly available at this point to protect anonymity of participants and to ensure data security until the TB & Tobacco project is completed. After project completion, selected data can be made available.

## Supplementary information

**Supplementary information** accompanies the paper on the *npj Primary Care Respiratory Medicine* website (10.1038/s41533-019-0146-6).

## Code availability

The codebook is included as additional information files.
