# Supplementary material for: Health worker and patient views on implementation of smoking cessation in routine tuberculosis care
Source: NPJ Prim Care Respir Med. 2019 Sep 3;29:34. doi: 10.1038/s41533-019-0146-6 (PMC6722140; doi:10.1038/s41533-019-0146-6)
Supplement: Supplementary file 1 — Supplementary Information. [file 41533_2019_146_MOESM1_ESM.pdf]

Supplementary Information:

Supplementary Methods 1: Interview guides for patients (English version)

Supplementary Methods 2: Interview guides for health workers (English version)

Supplementary Methods 3: Codebook for patient interviews

Supplementary Methods 4: Codebook for health worker interviews

Topic Guide for in-depth Interview: Patients receiving tobacco cessation counseling, facility level

Objectives of the interview:

- To examine whether the intervention was delivered as intended, including through description of the intervention as delivered
- To assess processes of implementation, including changes over time
- To explore intervention participants' responses to and perceptions of the intervention

Brief participant characteristics questionnaire to be filled in immediately before the interview or immediately after

Gender: ____________

Age: ________

Site ID:______________

Interviewer:__________

Date:__________

Time interview started:___________

Time interview completed:___________

Round 1 = Follow-up Day 5

This project has received funding from the European Union's Horizon 2020 research and innovation programme under grant agreement No 680995

Instructions for Interviewer:

The aim of the interview is to elicit as much information from the participant as possible on all aspects of the behaviour support, the implementation of the behaviour support package, their views on quitting tobacco, and their views on several aspects of our study. The following may help with getting the needed information:

- **Always give the participant sufficient time to think and to explain their thoughts. Even if the first question is a yes/no question to remind the participant of a situation, the following questions are more open. Do not quickly rephrase the question or give examples if the participants are silent at first. Give them a moment. Then, if a few minutes have passed and they have not given any answer, or asked you to clarify, you may rephrase the question.**

- **Always encourage the participant to say more and give more details. To do that there are some probes in the interview guides, marked in blue. Use these probes to encourage the participant to think about several aspects of the topic. If they mention points on their own, you do not need to use the probes. But make sure to always think about these dimensions of the topic and ask about them if the participant does not mention them on their own.**

- **Always ask questions like "Could you give some details? Could you tell me more? Why do you feel this way?" to really get into what the participant is saying. Do not move to the next question right after an answer was given. Instead, spend as much time as needed on one question, until you get no new information from the participant when you ask them to elaborate.**

- **Do not express your own views, or judge the participants, to avoid making them feel like they should say certain things to please you. There are no right or wrong answers here. We are interested in what the participants think, and whether that is the same as we are thinking ourselves is not relevant here. If you feel very uncomfortable with what is said, note it down and write it into the memo later.**

- **If you feel a participant feels too uncomfortable to answer a question, if possible skip ahead to a different question and ask the sensitive question again after a little while when the participant seems more relaxed or to trust you more. Remember of course that you should never force anybody to answer anything if they do not want to give an answer at all.**

- **Please always take notes about the specific interview situation directly after the interview. For instance: did somebody interrupt you? Was the place private, or did the participant have to fear somebody walking in on you? Did you both have enough time, or was there a need to rush? Did the participant freely attend the interview, or grudgingly? Was the family with them? With what expectations**

This project has received funding from the European Union's Horizon 2020 research and innovation programme under grant agreement No 680995

did you come to the interview yourself? Was it easy or difficult for you to interview this person, and why? All these and more info about the situation will help us in analysing the data later. Please use the general structure of the post-script memo guidance we have created, but feel free to add any additional points that you find important or interesting about the interview.

This project has received funding from the European Union's Horizon 2020 research and innovation programme under grant agreement No 680995

Welcome and thank you for making time for this interview today.

Purpose and Format of the Interview:

As you are aware, we are conducting an official study in collaboration with the National TB Programme on providing support for people with Tuberculosis (TB) who use tobacco products and wish to quit. You have chosen to participate in the study. We would like to ask you some questions about how satisfied you are with the programme, and are particularly interested in your views on the behavioural support, the messages you received from your health worker. This discussion will take around 30-45 minutes. We really appreciate your time. I would like to record this interview for the project, but will not use your name. If you agree, I will switch on the recorder now. If you want to stop the interview at any point, just let me know and we will stop immediately without negative consequences to you.

IMPLEMENTATION AND CONTEXT OF IMPLEMENTATION

Note to Interviewer: these first questions are important to make the patient feel safe talking to you, and to help them remember the session they took part in – all the following questions are about this particular session, so it is important that they are supported in remembering as much as they can.

Please always ask the general questions, then wait and see how the interviewee responds. Probe using the questions listed in bullet points below the main question. If interviewees bring up additional points, probe them on these new points as well.

If you remember, a few days ago you came in and saw a health worker here who talked to you about how to deal with illness. I would first like to ask you a few questions on how you feel about the discussion with the health worker.

1. Can you please describe the session you recently had with your health worker?*
    - What happened when you were sitting down with the health worker?
    - What did you talk about?
    - Was anybody there with you (a family member, a friend)?
    - Why do you think the health worker talked about these things?
    - What do you remember about materials the health worker used?

- How do you feel about how long the session with your health worker was?
    - How long do you think your health worker should talk to you during a session?

- How do you feel about the room/location where you talked to your health worker?*

    Ask about how they perceived the following items, amongst others:
    - noise,

This project has received funding from the European Union's Horizon 2020 research and innovation programme under grant agreement No 680995

- privacy,
- interruptions,
- distractions
- too hot, too cold,
- chairs or appropriate seating,
- posters or images on the wall,
- how was the lighting, was it a dark or light room/location,
- was the room outside or inside.

FLIP BOOK

So you were in the room with the health worker and talking about your health. I would like to ask you some more questions on what the health worker asked you or told you.

1. First, while you were talking, did your health worker show you any pictures on TB and health in a flip book during your session?
    - How did the health worker use this book during your session?
    Please describe what you remember about the session
        - Did the health worker show pictures and explain them, or just talked without referring to the images?
        - Was the health worker only talking, or did s/he also ask questions?
    - What do you remember about the messages on TB and health, what were they about?*
        - How do you feel about these messages?
        - How do you feel about following this advice?
        - What are the reasons you feel this way?

2. Did the health worker show you pictures on tobacco and smoking during your session?*
(if no: probe again what messages patient remembers)
    - What do you remember about these messages on tobacco and TB?
    - How do you feel about these tobacco messages?
    - How do you feel about following this advice?
    - What messages are most useful to your life?
    - What are the reasons you feel this way?

TOBACCO USE CONVERSATION

As I already mentioned, we are doing research on the different ways TB patients use tobacco, and on how health workers at TB clinics can offer help if somebody wants to stop using tobacco.

3. Did the health worker ask you whether you used tobacco while you were sitting together and looked at the flip book? Did they ask you at any point while you were seeing them?*
    - How did you feel about being asked whether you smoke or use tobacco?

This project has received funding from the European Union's Horizon 2020 research and innovation programme under grant agreement No 680995

- Was it something easy to talk about with the health worker?
- Why/why not?
- If it was difficult to talk about tobacco use, how did the health worker make you feel comfortable?/what could have made it easier?
- If it was easy, why was the health worker easy to talk to?

4. How do you feel about quitting tobacco right now?*
    - Will it be easy/difficult to quit?
        - Why/why not?

      Ask about

        - tobacco use in their families,
        - among their friends,
        - at work,
        - in social situations
        - afraid of quitting or side effects
        - not knowing how to quit
        - using tobacco inhibits feeling hungry/triggers weight loss
        - using tobacco to not feel hungry if not enough money to eat regularly
        - tobacco easy to get
    - What would help you quit tobacco right now?
      Ask about:
        - Family and friend support
        - Strict no tobacco rules at work
        - No tobacco in house
        - Laws
        - More expensive tobacco products
        - Less stress
        - Support from clinic
        - Medication

    - Is tobacco use something you can discuss with your friends/family?
    - Can you get support from friends/family?
        - Why/why not?
    - What do you believe will happen/change in your life if you stop using tobacco now?

MATERIALS

I would now like to ask you some questions on the materials on tobacco that you may have received from your health worker.

5. Did the health worker give you a leaflet after or during your session? (if no, probe: did the health worker ask you whether you are able to read?)
    - Can you describe the leaflet? What was it about?

This project has received funding from the European Union's Horizon 2020 research and innovation programme under grant agreement No 680995

- Probe: do you remember specific messages on tobacco?

- What do you think about the leaflet you received?
- What do you think about having a leaflet to take home with you after the session?
- What did you like or dislike about the leaflet?
- In what ways was the leaflet helpful?

How did you use the leaflet?

- Did you read it or look at pictures, share with somebody, have somebody read it to you, put it away?

6. Were there posters in the facility advertising tobacco cessation?
    - Can you describe what the posters were about?
    - Can you describe what you thought about them?
    - Why did you feel this way about the posters?

TRIAL AND CYTISINE

After the session, the health worker told you about the study and about maybe taking part in it. Then you talked to the researcher at the facility and decided to participate in this study. My next question is on your experience with the study so far.

7. When you went to speak with the researcher at the clinic, what did they explain to you about how the study works?

    listen for/probe specifically if interviewee doesn't bring it up:
    - Did somebody explain to you that you might be getting an inactive medicine?
    - If it does not come up, ask: what did they tell you about the medication to help you stop using tobacco that you are getting?

RECOMMENDATIONS

Before we conclude, we would like to get some advice from you on how to improve the session with your health worker in the future.

8. What do you think was good about the session with the flip book you had with your health worker?*
9. What do you think could be improved about the session with the flip book you had with your health worker?*
10. Is there anything else you would like to say?

Briefly summarize main points, get feedback on whether you captured the key points and clarify anything you are unsure of.

This project has received funding from the European Union's Horizon 2020 research and innovation programme under grant agreement No 680995

Thank you very much for your time today. Your feedback helps us to learn more about how to make this programme better. This is the end of our discussion. I will now turn off the recorder.

This project has received funding from the European Union's Horizon 2020 research and innovation programme under grant agreement No 680995

Topic Guide for in-depth Interview: Patients receiving tobacco cessation counseling, facility level

Objectives of this interview:

- To assess processes of implementation, including changes over time
- To explore intervention participants' responses to and perceptions of the intervention, including its effects on quit attempt

Brief participant characteristics questionnaire to be filled in immediately before the interview or immediately after

Gender: ____________

Age: ________

Site ID:______________

Interviewer:__________

Date:__________

Time interview starts:___________

Time interview completed:___________

Round 2: Follow-up Week 5

This project has received funding from the European Union's Horizon 2020 research and innovation programme under grant agreement No 680995

Instructions for Interviewer:

The aim of the interview is to elicit as much information from the participant as possible on all aspects of the behaviour support, the implementation of the behaviour support package, their views on quitting tobacco, and their views on several aspects of our study. The following may help with getting the needed information:

- **Always give the participant sufficient time to think and to explain their thoughts. Even if the first question is a yes/no question to remind the participant of a situation, the following questions are more open. Do not quickly rephrase the question or give examples if the participants are silent at first. Give them a moment. Then, if a few minutes have passed and they have not given any answer, or asked you to clarify, you may rephrase the question.**

- **Always encourage the participant to say more and give more details. To do that there are some probes in the interview guides, marked in blue. Use these probes to encourage the participant to think about several aspects of the topic. If they mention points on their own, you do not need to use the probes. But make sure to always think about these dimensions of the topic and ask about them if the participant does not mention them on their own.**

- **Always ask questions like "Could you give some details? Could you tell me more? Why do you feel this way?" to really get into what the participant is saying. Do not move to the next question right after an answer was given. Instead, spend as much time as needed on one question, until you get no new information from the participant when you ask them to elaborate.**

- **Do not express your own views, or judge the participants, to avoid making them feel like they should say certain things to please you. There are no right or wrong answers here. We are interested in what the participants think, and whether that is the same as we are thinking ourselves is not relevant here. If you feel very uncomfortable with what is said, note it down and write it into the memo later.**

- **If you feel a participant feels too uncomfortable to answer a question, if possible skip ahead to a different question and ask the sensitive question again after a little while when the participant seems more relaxed or to trust you more. Remember of course that you should never force anybody to answer anything if they do not want to give an answer at all.**

This project has received funding from the European Union's Horizon 2020 research and innovation programme under grant agreement No 680995

- **Please always take notes about the specific interview situation directly after the interview. For instance: did somebody interrupt you? Was the place private, or did the participant have to fear somebody walking in on you? Did you both have enough time, or was there a need to rush? Did the participant freely attend the interview, or grudgingly? Was the family with them? With what expectations did you come to the interview yourself? Was it easy or difficult for you to interview this person, and why? All these and more info about the situation will help us in analysing the data later. Please use the general structure of the post-script memo guidance we have created, but feel free to add any additional points that you find important or interesting about the interview.**

This project has received funding from the European Union's Horizon 2020 research and innovation programme under grant agreement No 680995

Welcome and thank you for making time for this interview today.

Purpose and Format of the Interview:

As you are aware, we are conducting an official study in collaboration with the National TB Programme on providing support for people with Tuberculosis (TB) who use tobacco products and wish to quit. You have chosen to participate in the study. We talked to you before a few weeks ago. Your answers were really helpful for us. This time we would like to ask you some more questions on how you have been in the past weeks, and on how satisfied you are with the advice your health worker gave you. This discussion will take around 20-30 minutes. We really appreciate your time. I would like to record this interview for the project, but will not use your name. If you agree, I will switch on the recorder now. If you want to stop the interview at any point, just let me know and we will stop immediately without negative consequences to you.

Note to Interviewer: these first questions are important to make the patient feel safe talking to you, and to help them remember the session they took part in – all the following questions are about this particular session, so it is important that they are supported in remembering as much as they can.

Please always ask the general questions, then wait and see how the interviewee responds. Probe using the questions listed in bullet points below the main question. If interviewees bring up additional points, probe them on these new points as well.

First, I would like to talk to you about your experiences with tobacco after your last session with the health worker, where you talked about TB, tobacco and health.

You might remember that in your session the health worker proposed to stop using tobacco and to set a quit day. That means a day after which you no longer use any tobacco.

Quit attempt

1. Have you talked to any of your friends or family about quitting tobacco after the session here at the clinic?*

    - Did you discuss TB with your family or friends as a reason to quit tobacco?

- Have you set a quit date with your health worker and tried to quit tobacco?
  [If yes: please ask all the questions below:] [If no, please skip and move to question 2]

- Could you please describe your quit attempt?
    - Did you manage to quit tobacco on the quit day you decided on with your health worker?

- Could you please describe the situations where you found/find it difficult to not use tobacco?
  - What do you think made it difficult to stop tobacco?
    - Friends/family

This project has received funding from the European Union's Horizon 2020 research and innovation programme under grant agreement No 680995

    - Tobacco sold near home or work or clinic
    - Withdrawal symptoms
    - Tobacco at workplace or at home
- Does anybody else in your family currently smoke?
  - If yes: How do you feel about not using tobacco around them?
    - ask regarding temptation, craving and peer pressure for using tobacco
- Are people tobacco/using tobacco at your work place? (if currently not working because of TB illness, ask about friends instead. If yes, probe with question)
  - How do you feel about not using tobacco around them?
    - ask regarding temptation, craving and peer pressure for using tobacco

- How do your friends react when you do not smoke when you're with them?
  - How do you feel about not using tobacco around your friends?
    - ask regarding temptation, craving and peer pressure for using tobacco

- How did you feel when you quit tobacco?
- What symptoms did you feel?
- What actions did you take to distract yourself from tobacco, e.g., chewing gum, clove, etc.?
- Who, if anyone, gave you suggestions on what actions to take?
- What did you do that was most helpful to not start using tobacco again? (probes: list messages from behavior support if no answer from patient)
-

2. **[if interviewee states to not have tried quitting at all:***
   - Why did you decide not to quit?
   - What do you think makes it difficult to stop tobacco?
     - Friends/family
     - Tobacco sold near home or work or clinic
     - Withdrawal symptoms
     - Tobacco at workplace or at home
   - Does anybody else in your family currently smoke?
     - If yes: How would you feel about not using tobacco around them?
       - ask regarding temptation, craving and peer pressure for using tobacco

   - Are people using tobacco at your work place? (if currently not working because of TB illness, ask about friends instead)
     - ask regarding temptation, craving and peer pressure for using tobacco

   - How do you feel about quitting tobacco now, today?]

This project has received funding from the European Union's Horizon 2020 research and innovation programme under grant agreement No 680995

Trial and cytisine incl adherence

After you talked to the health worker, you decided to participate in a study where you also receive some medication to quit tobacco, in addition to the TB medication. I would now like to ask you some questions about your experiences in this study and with the tobacco cessation medication.

Note to interviewer: at this point you can show the patient the photo of the tobacco cessation medication package and blister to help them remember which medication you are talking about. Careful: it doesn't matter whether they are in the treatment or control group, ask the question of any patient.

4. How do you feel about being offered both advice on how to quit tobacco and medication to help you quit tobacco?*

    - How do you feel about the medication you are taking to help you quit tobacco? (probe: How do you deal with side effects?)
    - How do you feel about the schedule for taking the medication?
        - easy to remember or complicated,
        - better to have one package,
        - difficult in combination with TB drugs….
    - Why is it easy or difficult to take all the medication?

- Do you feel the medication is useful for you in quitting tobacco?
    - In what way?
    - Why do you think that?

Recommendations

I would now like to ask you about your thoughts on what we could improve in this study on medication and advice to quit tobacco.

5. What do you think will help not start using tobacco again?*
    - support from friends
    - support from family
    - work place rules to not use tobacco
    - non-tobacco rules or laws in public places, such as restaurants, bars, streets, market places
    - feeling sick from TB
    - no money for tobacco/more expensive tobacco products…

6. What suggestions do you have for the study you are currently participating in?*
    - Is it easy to participate? Why/why not?
    - If it is a bit difficult, what would make it easier to participate?

This project has received funding from the European Union's Horizon 2020 research and innovation programme under grant agreement No 680995

7. Would you recommend the advice your health worker gave you to friends or family members who wish to stop using tobacco?
- Why or why not?
- Would you recommend the medication you are taking to stop using tobacco to your friends or family members? Why or why not?

8. Is there anything else you would like to say?

Briefly summarize main points, get feedback on whether you captured the key points and clarify anything you are unsure of.

Thank you very much for your time today. We need your feedback to learn more about how to make this programme better. This is the end of our discussion. I will now turn off the recorder.

This project has received funding from the European Union's Horizon 2020 research and innovation programme under grant agreement No 680995

Topic Guide for in-depth Interview: DOTS Facilitator, Facility Level

Objectives of the interview:

- To examine whether the intervention was delivered as intended, including through description of the intervention as delivered
- To assess processes of implementation, including changes over time
- To explore intervention deliverers' responses to and perceptions of the intervention

Brief participant characteristics questionnaire to be filled in immediately before the interview

Gender: ____________

Age: ________

How long have you been working at this center/institution? ________ years/months

How long have you been working with TB patients? _____ years/months

Site:_____________

Interviewer:____________

Date:__________

Time interview starts:__________

Time interview completed:______________

Round 1

Interview guide Health Workers/DOTS Facilitators Round 1 v2 13.03.17
This project has received funding from the European Union's Horizon 2020 research and innovation programme under grant agreement No 680995

Instructions for Interviewer:

The aim of the interview is to elicit as much information from the participant as possible on all aspects of the behaviour support, the implementation of the behaviour support package into their work days, and their views on several aspects of our study. The following may help with getting the needed information:

- **Always give the participant sufficient time to think and to explain their thoughts. Even if the first question is a yes/no question to remind the participant of a situation, the following questions are more open. Do not quickly rephrase the question or give examples if the participants are silent at first. Give them a moment. Then, if a few minutes have passed and they have not given any answer, or asked you to clarify, you may rephrase the question.**

- **Always encourage the participant to say more and give more details. To do that there are some probes in the interview guides, marked in blue. Use these probes to encourage the participant to think about several aspects of the topic. If they mention points on their own, you do not need to use the probes. But make sure to always think about these dimensions of the topic and ask about them if the participant does not mention them on their own.**

- **Always ask questions like "Could you give some details? Could you tell me more? Why do you feel this way?" to really get into what the participant is saying. Do not move to the next question right after an answer was given. Instead, spend as much time as needed on one question, until you get no new information from the participant when you ask them to elaborate.**

- **Do not express your own views, or judge the participants, to avoid making them feel like they should say certain things to please you. There are no right or wrong answers here. We are interested in what the participants think, and whether that is the same as we are thinking ourselves is not relevant here. If you feel very uncomfortable with what is said, note it down and write it into the memo later.**

- **Please always take notes about the specific interview situation directly after the interview. For instance: did somebody interrupt you? Was the place private, or did the participant have to fear their supervisor walking in on you? Did you both have enough time, or was there a need to rush? Did the participant freely attend the interview, or grudgingly? With what expectations did you come to the interview yourself? Was it easy or difficult for you to interview this person, and why? All these and more info about the situation will help us in analysing the data later. Please use the general structure of the post-script memo guidance we have created, but feel free to add any additional points that you find important or interesting about the interview.**

This project has received funding from the European Union's Horizon 2020 research and innovation programme under grant agreement No 680995

Welcome and thank you for making time for this interview today.

Purpose and Format of the Interview:

As you are aware, we are conducting an official study in collaboration with the National TB Programme on providing support for people with Tuberculosis (TB) who use tobacco and wish to quit. You have been involved in this study as one of the DOTS facilitators at this facility. You were trained to use the flip book and give your patients advice on quitting tobacco. We would like to ask you some questions about the tobacco cessation service in your facility, and are particularly interested in your views on the behavioural support you delivered to help TB patients quit tobacco. This discussion will take around 45 minutes. We appreciate your time. I would like to record this interview for the project, but will not use your name. If you agree, I will switch on the recorder now. If you wish to stop the interview at any time, just let me know and we will stop without negative consequences for you.

General experience
I would like to first ask you about your thoughts on the behaviour support and the project overall.

1. How do you feel about the behaviour support programme?*
   - How do you feel about the programme being used in your facility?
     - Why do you feel this way?

Flip book
To deliver the behaviour support, you were given a brief training and provided with a flip book and some materials, such as the leaflet and posters for the clinic. I would like to ask you about your thoughts on these items.

1. Do you use the flip book with all new TB patients?
   - Can you please describe how you are using the flip book?*
   - With whom do you use it? Why/why not do you use it with some patients?
   - How do you feel about using the flip book?
     - How do you feel about the images?
     - How do you feel about the texts on the back: too much text, not enough text?
     - How do you feel about the size and dimensions of the book: is it easy to hold and use during a consultation?
   - How do you feel about the general health messages and TB advice in the flip book?
     - What is the reason for this?
   - How do you feel about the tobacco cessation messages in the flip book?
     - What is the reason for this?

2. How have patients responded to the flip book and the TB health messages?*
   - Are there any differences between men and women?
   - Are there any differences between patients from different backgrounds?
     - Rural/urban
     - Highly educated, not well educated

This project has received funding from the European Union's Horizon 2020 research and innovation programme under grant agreement No 680995

  - with different occupations
  - different castes/class/backgrounds
  - religion
  - of different age
  - with different illnesses other than TB (*pre-existing conditions*)
    - specific illnesses: who are hearing/visually impaired

3. How are you using the materials with patients who are illiterate?*
   - Can you describe what you do and say differently – if at all – from your other patients?
   - Do you use the flip book differently with men or women who cannot read?
   - Do you use it differently with people of different backgrounds who cannot read?

Perceived usefulness/effectiveness
Let us talk a bit more about tobacco and TB patients.

4. Do you always ask patients about their tobacco use during the counselling session?*
   - Why/why not do you ask certain patients?

5. When do you ask patients about their tobacco use?
   - How do you feel about asking TB patients about their tobacco use?
   - Does this differ between asking men and women?
   - Do you feel differently asking patients from different backgrounds?
     - Rural/urban
     - Highly educated, not well educated
     - with different occupations
     - different castes/class/backgrounds
     - religion
     - of different age
     - with different illnesses other than TB (*pre-existing conditions*)
   - How do you feel about speaking about tobacco yourself?

6. How have patients responded specifically to the tobacco information and advice you gave?*
   - Are there any differences between men and women?
   - Are there any differences between patients from different backgrounds?
     - Rural/urban
     - Highly educated, not well educated
     - with different occupations
     - different castes/class/backgrounds
     - religion
     - of different age
     - with different illnesses other than TB (*pre-existing conditions*)
   - Who would respond in this way?
     - Why would they respond in this way?
   - What have patients said about potential difficulties stopping tobacco? What were they worried about?
     - tobacco use in their families,

This project has received funding from the European Union's Horizon 2020 research and innovation programme under grant agreement No 680995

- among their friends,
- at work,
- in social situations
- afraid of quitting or side effects
- not knowing how to quit
- Who has spoken about difficulties, and why do you think they responded this way?
  - Are there differences between people of different
  - Gender
  - Rural/urban
  - Highly educated, not well educated
  - with different occupations
  - religion
  - different castes/class/backgrounds
  - of different age
  - with different illnesses other than TB (*pre-existing conditions*)

Leaflet
I would like to come back to the materials we have given you. What are your thoughts on the leaflet and the poster?

7. Do you give the leaflet to all patients during or after your counselling session?
   - Whom do you give it to? Why/why not do you give it to some patients?

   If not to all patients:
     - Are there differences between people of different backgrounds?
     - Gender
     - Rural/urban
     - Highly educated, not well educated
     - with different occupations
     - religion
     - different castes/class/backgrounds
     - of different age
     - with different illnesses other than TB (*pre-existing conditions*)

8. How do patients react when you offer them the leaflet?
   - Are there differences between men and women?
   - Are there differences between people of different backgrounds?
     - Rural/urban
     - Highly educated, not well educated
     - with different occupations
     - religion
     - different castes/class/backgrounds
     - of different age
     - with different illnesses other than TB (*pre-existing conditions*)
   - Which persons responded in this way to the leaflet?
   - Why do you think they respond in this way?

9. How do you use the leaflet with patients who cannot read?

This project has received funding from the European Union's Horizon 2020 research and innovation programme under grant agreement No 680995

- Do you explain the pictures?
- Read leaflet to them?
- Ask them to show to somebody to read at home?
- Not give out leaflet at all to those patients?

- Do you use the leaflet differently with men or women who cannot read?
- Do you use it differently with people of different backgrounds who cannot read?
    - Rural/urban
    - Highly educated, not well educated
    - with different occupations
    - religion
    - different castes/class/backgrounds
    - of different age
    - with different illnesses other than TB (*pre-existing conditions*)

10. How do you feel about the leaflet and its messages?
    - Why do you feel this way?

Poster

11. Is there a poster in your facility advertising the cessation services? (if yes, probe with question #12)

12. How do you feel about the poster and its messages?
    - Why do you feel this way?

13. Do patients talk to you about the poster?
    - Have you shown patients the poster/talked to patients about the poster?
    - Who talked to you about the poster? People of different
        - Gender
        - Rural/urban
        - Highly educated, not well educated
        - with different occupations
        - religion
        - different castes/class/backgrounds
        - of different age
        - with different illnesses other than TB (*pre-existing conditions*)?

COM-B related

I would now like to ask you a few questions on the training on flipbook and counselling you were offered by the TB&Tobacco project before starting this project and giving the TB & tobacco advice.

14. How do you feel about the training you received in delivering the behaviour support?
    - What kinds of information and materials have been made available to you? Did it help you to improve your knowledge and skills about delivering tobacco cessation support?
        - How specifically did it help/which aspects of it helped?
    - Do you feel the need for any additional training on knowledge or skills?
        - If yes, what training would you like to receive?

This project has received funding from the European Union's Horizon 2020 research and innovation programme under grant agreement No 680995

- Why would you like to receive this particular training?

Context
The programme on tobacco cessation takes place at your clinic. I would like to ask you some questions about your work environment and your work day in the clinic.

15. Have you asked any colleagues to also deliver the behaviour support, e.g. when you can't come to work?
    - If yes: could you please give us their names so we can interview them as well?
    - Have you given the project materials (flip book/leaflets/training materials) to these colleagues?
16. How do you feel about delivering the programme as part of your work day/routine?*
    - Why do you feel this way?
    - What about
        - Time
        - Space
        - Privacy
        - Supervisors/in-charge support
        - Length of flip book session
    - Can you please describe how you integrate the flip book session into your daily routine?
    - What changes in your daily practice, if any, do you plan on making in the next few months while you are delivering this behaviour support?

17. The programme is intended to support TB patients who wish to stop using tobacco. What do you think might affect a patient's ability to quit tobacco?*
    How about
    - other family members' tobacco habits,
    - price of tobacco products,
    - where tobacco is sold (nearby),
    - exposure to second hand tobacco at work, inside their home
    - Does this differ for men and women?
    - Does this differ for patients of different backgrounds?
        - Rural/urban
        - Highly educated, not well educated
        - with different occupations
        - religion
        - different castes/class/backgrounds
        - of different age
        - with different illnesses other than TB (*pre-existing conditions*)

18. Looking forward, what do you feel is needed to make tobacco cessation easier for patients?
    - Is this the same for all patients, or does it differ for patients from different backgrounds?
        - gender
        - Rural/urban
        - Highly educated, not well educated

This project has received funding from the European Union's Horizon 2020 research and innovation programme under grant agreement No 680995

- with different occupations
- religion
- different castes/class/backgrounds
- of different age
- with different illnesses other than TB (*pre-existing conditions*)

19. Is there anything else you'd like to say?

Briefly summarize main points, get feedback on whether you captured the key points and clarify anything you are unsure of.

Thank you very much for your time today. This is the end of our discussion. I will now turn off the recorder.

This project has received funding from the European Union's Horizon 2020 research and innovation programme under grant agreement No 680995

Topic Guide for in-depth Interview: DOTS Facilitator, Facility Level

Objectives of the interview:

- To examine whether the intervention was delivered as intended, including through description of the intervention as delivered
- To assess processes of implementation, including changes over time
- To explore intervention deliverers' responses to and perceptions of the intervention

Brief participant characteristics questionnaire to be filled in immediately before the interview

Gender: ____________

Age: ________

How long have you been working at this center/institution? ________ years/months

How long have you been working with TB patients? _____ years/months

Site:_____________

Interviewer:____________

Date:__________

Time interview starts:__________

Time interview completed:______________

Round 2

This project has received funding from the European Union's Horizon 2020 research and innovation programme under grant agreement No 680995

Instructions for Interviewer:

The aim of the interview is to elicit as much information from the participant as possible on all aspects of the behaviour support, the implementation of the behaviour support package into their work days, and their views on several aspects of our study. The following may help with getting the needed information:

- **Always give the participant sufficient time to think and to explain their thoughts. Even if the first question is a yes/no question to remind the participant of a situation, the following questions are more open. Do not quickly rephrase the question or give examples if the participants are silent at first. Give them a moment. Then, if a few minutes have passed and they have not given any answer, or asked you to clarify, you may rephrase the question.**

- **Always encourage the participant to say more and give more details. To do that there are some probes in the interview guides, marked in blue. Use these probes to encourage the participant to think about several aspects of the topic. If they mention points on their own, you do not need to use the probes. But make sure to always think about these dimensions of the topic and ask about them if the participant does not mention them on their own.**

- **Always ask questions like "Could you give some details? Could you tell me more? Why do you feel this way?" to really get into what the participant is saying. Do not move to the next question right after an answer was given. Instead, spend as much time as needed on one question, until you get no new information from the participant when you ask them to elaborate.**

- **Do not express your own views, or judge the participants, to avoid making them feel like they should say certain things to please you. There are no right or wrong answers here. We are interested in what the participants think, and whether that is the same as we are thinking ourselves is not relevant here. If you feel very uncomfortable with what is said, note it down and write it into the memo later.**

- **Please always take notes about the specific interview situation directly after the interview. For instance: did somebody interrupt you? Was the place private, or did the participant have to fear their supervisor walking in on you? Did you both have enough time, or was there a need to rush? Did the participant freely attend the interview, or grudgingly? With what expectations did you come to the interview yourself? Was it easy or difficult for you to interview this person, and why? All these and more info about the situation will help us in analysing the data later. Please use the general structure of the post-script memo guidance we have created, but feel free to add any additional points that you find important or interesting about the interview.**

This project has received funding from the European Union's Horizon 2020 research and innovation programme under grant agreement No 680995

Welcome and thank you for making time for this interview today.

Purpose and Format of the Interview:

As you are aware, we are conducting an official study in collaboration with the National TB Programme on providing support for people with Tuberculosis (TB) who smoke and wish to quit tobacco. You have been involved in this study as one of the DOTS facilitators at this facility. We already spoke to you a few months ago. Your answers were really helpful to us. We would like to ask you some more questions about the tobacco cessation service in your facility, and are particularly interested in your views on the behavioural support you delivered to help TB patients quit tobacco. This discussion will take around 45 minutes. We appreciate your time. I would like to record this interview for the project, but will not use your name. If you agree, I will switch on the recorder now. If you wish to stop the interview at any time, just let me know and we will stop without negative consequences for you.

General experience

I would like to first ask you again about your thoughts on the behaviour support and the project overall.

1. How do you feel about the behaviour support programme?
    - How do you feel about the programme being used in your facility?

To deliver the behaviour support, you were given brief training and provided with a flip book and some materials, such as the leaflet and posters for the clinic. I would like to ask you about your thoughts on these items.

Flip book

1. Do you still use the flip book with all new TB patients?
    - Can you please describe how you are using the flip book?*
    - With whom do you use it? Why/why not do you use it with some patients?
    - How do you feel about using the flip book?
        - How do you feel about the images?
        - How do you feel about the texts on the back: too much text, not enough text,
        - How do you feel about the size and dimensions of the book: is it easy to hold and use during a consultation?
    - How do you feel about the general health messages and TB advice in the flip book?
        - What is the reason for this?
    - How do you feel about the tobacco cessation messages in the flip book?*
        - What is the reason for this?

2. How have patients responded to the flip book and the TB health messages?*
    - Are there any differences between men and women?
    - Are there any differences between patients from different backgrounds?
        - Rural/urban
        - Highly educated, not well educated
        - with different occupations
        - different castes/class/backgrounds
        - religion

This project has received funding from the European Union's Horizon 2020 research and innovation programme under grant agreement No 680995

  - of different age
  - with different illnesses other than TB (*pre-existing conditions*)

3. How are you using the materials with patients who cannot read?
   - Can you describe what you do and say differently – if at all – from your other patients?
   - Do you use the flip book differently with men or women who cannot read?
   - Do you use it differently with people of different backgrounds who cannot read?

Perceived usefulness/effectiveness
Let us talk a bit more about tobacco and TB patients.

4. Do you always ask patients about their tobacco use during the counselling session?
   - Why/why not do you ask certain patients?
5. When do you ask patients about their tobacco use? How do you feel about asking TB patients about their tobacco use?*
   - Does this differ between asking men and women?
   - Do you feel differently asking patients from different backgrounds?
     - Rural/urban
     - Highly educated, not well educated
     - with different occupations
     - different castes/class/backgrounds
     - religion
     - of different age
     - with different illnesses other than TB (*pre-existing conditions*)
   - How do you feel about speaking about tobacco yourself?

6. How have patients responded specifically to the tobacco information and advice you gave?*
   - Are there any differences between men and women?
   - Are there any differences between patients from different backgrounds?
     - Rural/urban
     - Highly educated, not well educated
     - with different occupations
     - different castes/class/backgrounds
     - religion
     - of different age
     - with different illnesses other than TB (*pre-existing conditions*)

   Who would respond in this way?
     - Why would they respond in this way?
   - What have patients said about potential difficulties stopping tobacco?
   What were they worried about?
     - tobacco use in their families,
     - among their friends,
     - at work,
     - in social situations
     - afraid of quitting or side effects
     - not knowing how to quit

This project has received funding from the European Union's Horizon 2020 research and innovation programme under grant agreement No 680995

- Who has spoken about difficulties, and why do you think they responded this way?
  - Are there differences between people of different
  - Gender
  - Rural/urban
  - Highly educated, not well educated
  - with different occupations
  - religion
  - different castes/class/backgrounds
  - of different age
  - with different illnesses other than TB (*pre-existing conditions*)

7. How essential is the tobacco cessation programme to meet the needs of TB patients?
   - How will it fill a gap, do you see a gap for TB patients?

Cytisine and Trial
As you know, there is also a study here at your clinic where patients receive medication to help them quit using tobacco. I would like to hear your thoughts on this study.

8. How have patients so far experienced using the drug cytisine to help them quit tobacco? What have they told you when they came back to speak with you?*
   - Are there any differences between men and women?
   - Are there any differences between people of different backgrounds?
   - Rural/urban
   - Highly educated, not well educated
   - with different occupations
   - religion
   - different castes/class/backgrounds
   - of different age
   - with different illnesses other than TB (*pre-existing conditions*)

8. What do you think about having patients use the drug to help them quit tobacco?
   - Can you think of any disadvantages for patients using the drug?
- Does this differ between men and women?
- Does this differ between people of different backgrounds?
  - Rural/urban
  - Highly educated, not well educated
  - with different occupations
  - religion
  - different castes/class/backgrounds
  - of different age
  - with different illnesses other than TB (*pre-existing conditions*)

- How do you feel about the trial, that some patients are included in the trial and some are not?
  - Why do you feel this way?

Context
I have a few final questions on your work day and the work environment.

This project has received funding from the European Union's Horizon 2020 research and innovation programme under grant agreement No 680995

9. Have you asked any other or new colleagues to also deliver the behaviour support, e.g. when you can't come to work?
   If yes: could you please give us their names so we can interview them as well?

10. Could you please describe any changes to your clinic since you started the project?*
    - How well does the programme fit with your clinic's work day now?
    - How do you juggle competing priorities in your daily work with TB patients?

      Probes for changes:
      - New rules about tobacco use at clinic
      - New signs
      - New posters
      - Different responsibilities
      - Other space now used for consultation

11. Who do you ask if you have questions about the programme or on how to use it in your work routine?
    - How available are these individuals?
    - Do you talk to your colleagues about the programme and give each other advice?

12. Looking forward, what do you feel is needed to make tobacco cessation easier for patients?*
    - Is this the same for all patients, or does it differ for patients from different backgrounds?

      Ask about:
      - Family support
      - Workplace rules
      - No smoking laws
      - Price of tobacco products
      - Tobacco shops nearby
      - Using tobacco to not gain weight
      - Using tobacco to not feel hungry if not enough money to eat regularly…

13. Is there anything else you'd like to say?

Briefly summarize main points, get feedback on whether you captured the key points and clarify anything you are unsure of.

Thank you very much for your time today. This is the end of our discussion. I will now turn off the recorder.

This project has received funding from the European Union's Horizon 2020 research and innovation programme under grant agreement No 680995

Topic Guide for in-depth Interview: DOTS Facilitator, Facility Level

Objectives of the interview:

- To examine whether the intervention was delivered as intended, including through description of the intervention as delivered
- To assess processes of implementation, including changes over time
- To explore intervention deliverers' responses to and perceptions of the intervention

Brief participant characteristics questionnaire to be filled in immediately before the interview

Gender: ____________

Age: ________

How long have you been working at this center/institution? ________ years/months

How long have you been working with TB patients? _____ years/months

What is your education?

Site:_____________

Interviewer:____________

Date:__________

Time interview starts:__________

Time interview completed:______________

Round 3

This project has received funding from the European Union's Horizon 2020 research and innovation programme under grant agreement No 680995

Instructions for Interviewer:

The aim of the interview is to elicit as much information from the participant as possible on all aspects of the behaviour support, the implementation of the behaviour support package into their work days, and their views on several aspects of our study. The following may help with getting the needed information:

- **Always give the participant sufficient time to think and to explain their thoughts. Even if the first question is a yes/no question to remind the participant of a situation, the following questions are more open. Do not quickly rephrase the question or give examples if the participants are silent at first. Give them a moment. Then, if a few minutes have passed and they have not given any answer, or asked you to clarify, you may rephrase the question.**

- **Always encourage the participant to say more and give more details. To do that there are some probes in the interview guides, marked in blue. Use these probes to encourage the participant to think about several aspects of the topic. If they mention points on their own, you do not need to use the probes. But make sure to always think about these dimensions of the topic and ask about them if the participant does not mention them on their own.**

- **Always ask questions like "Could you give some details? Could you tell me more? Why do you feel this way?" to really get into what the participant is saying. Do not move to the next question right after an answer was given. Instead, spend as much time as needed on one question, until you get no new information from the participant when you ask them to elaborate.**

- **Do not express your own views, or judge the participants, to avoid making them feel like they should say certain things to please you. There are no right or wrong answers here. We are interested in what the participants think, and whether that is the same as we are thinking ourselves is not relevant here. If you feel very uncomfortable with what is said, note it down and write it into the memo later.**

- **Please always take notes about the specific interview situation directly after the interview. For instance: did somebody interrupt you? Was the place private, or did the participant have to fear their supervisor walking in on you? Did you both have enough time, or was there a need to rush? Did the participant freely attend the interview, or grudgingly? With what expectations did you come to the interview yourself? Was it easy or difficult for you to interview this person, and why? All these and more info about the situation will help us in analysing the data later. Please use the general structure of the post-script memo guidance we have created, but feel free to add any additional points that you find important or interesting about the interview.**

This project has received funding from the European Union's Horizon 2020 research and innovation programme under grant agreement No 680995

Welcome and thank you for making time for this interview today.

Purpose and Format of the Interview:

As you are aware, we are conducting an official study in collaboration with the National TB Programme on providing support for people with Tuberculosis (TB) who smoke and wish to quit tobacco. You have been involved in this study as one of the DOTS facilitators at this facility. We already spoke to you twice to keep updated on your views about the programme. This will be our last interview, so we would like to ask you some questions about the tobacco cessation service in your facility at this point in the programme, and are particularly interested in your views on the behavioural support you delivered to help TB patients quit tobacco. This discussion will take around 45 minutes. We appreciate your time. I would like to record this interview for the project, but will not use your name. If you agree, I will switch on the recorder now. If you wish to stop the interview at any time, just let me know and we will stop without negative consequences for you.

1. **I would like you to describe to me a specific situation with a patient in the past 6 months that really made an impact on you, that you remember very well. Anything related to your daily work. I am interested in everything.**

COM-B related

You have now been delivering tobacco cessation advice to a lot of patients. We would really like to talk to you about how you feel about delivering this and the TB advice.

2. What do you think about continuing to inform patients about TB and tobacco cessation after this project ends?*
    - Why do you think this?
    - What do you think you will need to be able to continue giving these messages?
        - To all patients or to some of your patients?
        - Which groups of patients will you be able to provide the behaviour support to?
    - From whom will you need support? Why from these people?
    - Have you received any feedback on your work on tobacco cessation (outside the research team)?
        - From whom?
        - How often?
        - Where: a special meeting, or during the day?
        - Beyond you're your clinic supervisors, do you receive any feedback or information from district level NTP about your work?
    - What kinds of things might affect continuing this tobacco cessation programme in your facility in the long run?
        - Why do you think these could affect the programme?
        - How could these be addressed?

This project has received funding from the European Union's Horizon 2020 research and innovation programme under grant agreement No 680995

Context
I would like to ask you a question about your work environment and your work day.

3. What do you think has changed around here since the project started?
   - New rules about not using tobacco for employees
   - new no tobacco signs
   - people talking about tobacco at work
   - new posters or images

Why do you think these changes occurred?

Patient rapport
As part of your work, you talk to patients a lot, and you support them. We would like to learn more about how to work well together with patients and would like to get your advice.

3. Can you please describe how you communicated with your patients? How do you make them feel comfortable so they would talk to you?
   - Can you please describe how you interacted with patients of different backgrounds?
     - Gender
     - Rural/urban
     - Highly educated, not well educated
     - with different occupations
     - religion
     - different castes/class/backgrounds
     - of different age
     - with different illnesses other than TB (*pre-existing conditions*)

4. From your experience as a health professional, do you have any recommendations on how to best create a situation in which patients can speak openly about their illness and their tobacco habits? What would you tell other health workers?
   - What – if anything – are you doing differently with current patients than with the very first patients you delivered the support to?
   - Time spent on each patient
   - Asking more questions
   - Presenting certain slides more quickly than others
   - Talk differently to different patients
   - Prioritizing some messages
   - Why are you doing these things differently now?

5. Is there anything else you would like to say?

Briefly summarize main points, get feedback on whether you captured the key points and clarify anything you are unsure of.

Thank you very much for your time today. This is the end of our discussion. I will now turn off the recorder.

This project has received funding from the European Union's Horizon 2020 research and innovation programme under grant agreement No 680995

Supplementary Methods 3: Codebook for patient interviews

Notes:

- Theoretical framework = Theoretical Domains Framework [1]
- Code both positive and negative responses
- Emerging constructs are allowed

Domain and constructs	Definitions
TDF01 Knowledge	An awareness of the existence of something
TDF1.1 Knowledge	An awareness of the existence of something (including knowledge of condition/scientific rationale)
TDF1.2 Procedural knowledge	Knowing how to do something
TDF1.3 Knowledge of task environment	Knowledge of the social and material context in which a task is undertaken
TDF02 Skills	An ability or proficiency acquired through practice
TDF2.1 Skills	An ability or proficiency acquired through training and/or practice
TDF2.2 Skills development	The gradual acquisition or advancement through progressive stages of an ability or proficiency acquired through training and practice
TDF2.3 Competence	One's repertoire of skills, and ability especially as it is applied to a task or set of tasks
TDF2.4 Ability	Competence or capacity to perform a physical or mental act. Ability may be either unlearned or acquired by education and practice
TDF2.5 Interpersonal skills	An aptitude enabling a person to carry on effective relationships with others, such as an ability to cooperate, to assume appropriate social responsibilities or to exhibit adequate flexibility

TDF2.6 Practice	Repetition of an act, behaviour, or series of activities, often to improve performance or acquire a skill
TDF2.7 Skill assessment	A judgment of the quality, worth, importance, level, or value of an ability or proficiency acquired through training and practice
TDF03 Social role and identity	A coherent set of behaviours and displayed personal qualities of an individual in a social or work setting. We will only look for social role, not professional role in patients
TDF3.2 Professional role	The behaviour considered appropriate for a particular kind of work or social position
TDF3.3 Social identity	The set of behavioural or personal characteristics by which an individual is recognizable [and portrays] as a member of a social group
TDF3.4 Identity	An individual's sense of self defined by a) a set of physical and psychological characteristics that is not wholly shared with any other person and b) a range of social and interpersonal affiliations (e.g., ethnicity) and social roles
TDF3.5 Professional boundaries	The bounds or limits relating to, or connected with a particular profession or calling
TDF3.7 Group identity	The set of behavioural or personal characteristics by which an individual is recognizable [and portrays] as a member of a group
TDF04 Beliefs about capabilities	Acceptance of the truth, reality, or validity about an ability, talent, or facility that a person can put to constructive use
TDF4.1 Self-Confidence	Self-assurance or trust in one's own abilities, capabilities and judgment
TDF4.2 Perceived competence	An individual's belief in his or her ability to learn and execute skills
TDF4.3 Self-Efficacy	An individual's capacity to act effectively to bring about desired results, as perceived by the individual
TDF4.4 Perceived behavioural control	An individual's perception of the ease or difficulty of performing the behaviour of interest
TDF4.5 Beliefs	The thing believed; the proposition or set of propositions held true

TDF4.7 Empowerment	The promotion of the skills, knowledge and confidence necessary to take great control of one's life as in certain educational or social schemes; the delegation of increased decision-making powers to individuals or groups in a society or organisation
TDF4.8 Professional confidence	An individual's belief in his or her repertoire of skills, and ability especially as it is applied to a task or set of tasks
TDF05 Optimism	The confidence that things will happen for the best or that desired goals will be attained
TDF5.1 Optimism	The attitude that outcomes will be positive and that people's wishes or aims will ultimately be fulfilled
TDF5.2 Pessimism	The attitude that things will go wrong and that people's wishes or aims are unlikely to be fulfilled
TDF5.3 Unrealistic optimism	The inert tendency for humans to over-rate their own abilities and chances of positive outcomes compared to those of other people
TDF06 Beliefs about Consequences	Acceptance of the truth, reality, or validity about outcomes of a behaviour in a given situation
TDF6.1 Beliefs	The thing believed; the proposition or set of propositions held true
TDF6.2 Outcome expectancies	Cognitive, emotional, behavioural, and affective outcomes that are assumed to be associated with future or intended behaviours. These assumed outcomes can either promote or inhibit future behaviours
TDF6.3 Characteristics of outcome expectancies	Characteristics of the cognitive, emotional and behavioural outcomes that individuals believe are associated with future or intended behaviours and that are believed to either promote or inhibit these behaviours. These include whether they are sanctions/rewards, proximal/distal, valued/not valued, probable/improbable, salient/not salient, perceived risks or threats
TDF6.4 Anticipated regret	A sense of the potential negative consequences of a decision that influences the choice made: for example an individual may decide not to make an investment because of the feelings associated with an imagined loss
TDF6.5 Consequents	An outcome of behaviour in a given situation
TDF07 Reinforcement	Increasing the probability of a response by arranging a dependent relationship, or contingency, between the response and a given stimulus

TDF7.1 Rewards (proximal; distal, valued; not valued; probable; improbable)	Return or recompense made to, or received by a person contingent on some performance
TDF7.2 Incentives	An external stimulus, such as condition or object, that enhances or serves as a motive for behaviour
TDF7.3 Punishment	The process in which the relationship between a response and some stimulus or circumstance results in the response becoming less probable; a painful, unwanted or undesired event or circumstance imposed as a penalty on a wrongdoer
TDF7.4 Consequents	An outcome of behaviour in a given situation
TDF7.5 Reinforcement	A process in which the frequency of a response is increased by a dependent relationship or contingency with a stimulus
TDF7.6 Contingencies	A conditional probabilistic relation between two events. Contingencies may be arranged via dependencies or they may emerge by accident
TDF08 Intentions	A conscious decision to perform a behaviour or a resolve to act in a certain way
TDF8.1 Stability of intentions	Ability of one's resolve to remain in spite of disturbing influences.
TDF09 Goals	Mental representations of outcomes or end states that an individual wants to achieve
TDF9.5 Action planning	The action or process of forming a plan regarding a thing to be done or a deed.
TDF9.6 Implementation intention	The plan that one creates in advance of when, where and how one will enact a behaviour.
TDF10 Memory, Attention and Decision Processes	The ability to retain information, focus selectively on aspects of the environment and choose between two or more alternatives
TDF10.1 Memory	The ability to retain information or a representation of a past experience, based on the mental processes of learning or encoding retention across some interval of time, and retrieval or reactivation of the memory; specific information of a specific past.

TDF10.2 Attention	A state of awareness in which the senses are focussed selectively on aspects of the environment and the central nervous system is in a state of readiness to respond to stimuli.
TDF10.3 Attention control	The extent to which a person can concentrate on relevant cues and ignore all irrelevant cues in a given situation.
TDF10.4 Decision making	The cognitive process of choosing between two or more alternatives, ranging from the relatively clear cut to the complex.
TDF10.5 Cognitive overload or tiredness	The situation in which the demands placed on a person by mental work are greater than a person's mental abilities.
TDF11 Environmental Context and Resources	Any circumstance of a person's situation or environment that discourages or encourages the development of skills and abilities, independence, social competence, and adaptive behaviour
TDF11.1 Environmental stressors	External factors in the environment that cause stress.
TDF11.2 Resources_material resources	Commodities and human resources used in enacting a behaviour.
TDF11.3 Organisational culture and climate	A distinctive pattern of thought and behaviour shared by members of the same organisation and reflected in their language, values, attitudes, beliefs and customs.
TDF11.4 Salient events or critical incidents	Occurrences that one judges to be distinctive, prominent or otherwise significant.
TDF11.5 Person x environment interaction	Interplay between the individual and their surroundings.
TDF11.6 Barriers and facilitators	In psychological contexts barriers/facilitators are mental, emotional or behavioural limitations/strengths in individuals or groups.
TDF12 Social influences	Those interpersonal processes that can cause individuals to change their thoughts, feelings, or behaviours
TDF12.01 Social pressure	The exertion of influence on a person or group by another person or group.
TDF12.02 Social norms	Socially determined consensual standards that indicate a) what behaviours are considered typical in a given context and b) what behaviours are considered proper in the context.

TDF12.03 Group conformity	The act of consciously maintaining a certain degree of similarity to those in your general social circles.
TDF12.05 Group norms	Any behaviour, belief, attitude or emotional reaction held to be correct or acceptable by a given group in society.
TDF12.06 Social support	The apperception or provision of assistance or comfort to others, typically in order to help them cope with a variety of biological, psychological and social stressors. Support may arise from any interpersonal relationship in an individual's social network, involving friends, neighbours, religious institutions, colleagues, caregivers or support groups.
TDF12.07 Power	The capacity to influence others, even when they try to resist this influence.
TDF12.08 Intergroup conflict	Disagreement or confrontation between two or more groups and their members. This may involve physical violence, interpersonal discord, or psychological tension.
TDF12.09 Alienation	Estrangement from one's social group; a deep seated sense of dissatisfaction with one's personal experiences that can be a source of lack of trust in one's social or physical environment or in oneself; the experience of separation between thoughts and feelings.
TDF12.10 Group identity	The set of behavioural or personal characteristics by which an individual is recognizable [and portrays] as a member of a group.
TDF12.11 Modelling	In developmental psychology the process in which one or more individuals or other entities serve as examples (models) that a child will copy.
TDF13 Emotion	A complex reaction pattern, involving experiential, behavioural, and physiological elements, by which the individual attempts to deal with a personally significant matter or event
TDF13.1 Fear	An intense emotion aroused by the detection of imminent threat, involving an immediate alarm reaction that mobilises the organism by triggering a set of physiological changes.
TDF13.2 Anxiety	A mood state characterised by apprehension and somatic symptoms of tension in which an individual anticipates impending danger, catastrophe or misfortune.
TDF13.4 Stress	A state of physiological or psychological response to internal or external stressors.

TDF13.5 Depression	A mental state that presents with depressed mood, loss of interest or pleasure, feelings of guilt or low self-worth, disturbed sleep or appetite, low energy, and poor concentration.
TDF13.7 Burn-out	Physical, emotional or mental exhaustion, especially in one's job or career, accompanied by decreased motivation, lowered performance and negative attitudes towards oneself and others.
TDF14 Behavioural Regulation	Anything aimed at managing or changing objectively observed or measured actions
TDF14.1 Self-monitoring	A method used in behavioural management in which individuals keep a record of their behaviour, especially in connection with efforts to change or regulate the self; a personality trait reflecting an ability to modify one's behaviour in response to situation.
TDF14.2 Breaking habit	To discontinue a behaviour or sequence of behaviours that is automatically activated by relevant situational cues.
TDF14.3 Action planning	The action or process of forming a plan regarding a thing to be done or a deed.
EMERGING CONSTRUCTS	
Therapy procedure/Medicine for TB	For descriptions of how their disease was detected, what services or care they received at the clinic, the process of coming to the clinic for the first time
Faith/Religious beliefs	Expressions of faith in the context of TB disease, treatment, cessation, or social contexts of the interview participants
Perceptions of the TB & Tobacco Intervention	Descriptions of what participants remember about the behaviour support and intervention overall, including cytisine, as well as their opinions about it
Smoking behaviour	Descriptions of smoking behaviour that who do not fit any of the other constructs

1. Cane J, O’Connor D, Michie S, Newman M, Papadopoulous L, Sigsworth J, et al. Validation of the theoretical domains framework for use in behaviour change and implementation research. Implement. Sci. 2012;7:37. Available from: http://implementationscience.biomedcentral.com/articles/10.1186/1748-5908-7-37

Supplementary Methods 4: Codebook for health worker interviews

Notes:

- Theoretical Framework = Consolidated Framework for Implementation Research (CFIR) [1], see also www.cfir.org
- Code both positive and negative responses
- Emerging constructs are allowed

Construct		Definition of the CFIR
I. INTERVENTION CHARACTERISTICS		
A	Intervention Source	Perception of key stakeholders about whether the intervention is externally or internally developed.
B	Evidence Strength & Quality	Stakeholders' perceptions of the quality and validity of evidence supporting the belief that the intervention will have desired outcomes.
C	Relative Advantage	Stakeholders' perception of the advantage of implementing the intervention versus an alternative solution.
D	Adaptability	The degree to which an intervention can be adapted, tailored, refined, or reinvented to meet local needs.
F	Complexity	Perceived difficulty of implementation, reflected by duration, scope, radicalness, disruptiveness, centrality, and intricacy and number of steps required to implement.
G	Design Quality & Packaging	Perceived excellence in how the intervention is bundled, presented, and assembled.
H	Cost	Costs of the intervention and costs associated with implementing the intervention including investment, supply, and opportunity costs.
II. OUTER SETTING		
A	Patient Needs & Resources	The extent to which patient needs, as well as barriers and facilitators to meet those needs, are accurately known and prioritized by the organization.
C	Peer Pressure	Mimetic or competitive pressure to implement an intervention; typically because most or other key peer or competing organizations have already implemented or are in a bid for a competitive edge.
D	External Policy & Incentives	A broad construct that includes external strategies to spread interventions, including policy and regulations (governmental or other central entity), external mandates, recommendations and guidelines, pay-for-performance, collaboratives, and public or benchmark reporting.

III. INNER SETTING		
A	Structural Characteristics	The social architecture, age, maturity, and size of an organization.
B	Networks & Communications	The nature and quality of webs of social networks and the nature and quality of formal and informal communications within an organization.
C	Culture	Norms, values, and basic assumptions of a given organization.
D	Implementation Climate	The absorptive capacity for change, shared receptivity of involved individuals to an intervention, and the extent to which use of that intervention will be rewarded, supported, and expected within their organization.
1	Tension for Change	The degree to which stakeholders perceive the current situation as intolerable or needing change.
2	Compatibility	The degree of tangible fit between meaning and values attached to the intervention by involved individuals, how those align with individuals' own norms, values, and perceived risks and needs, and how the intervention fits with existing workflows and systems.
3	Relative Priority	Individuals' shared perception of the importance of the implementation within the organization.
4	Organizational Incentives & Rewards	Extrinsic incentives such as goal-sharing awards, performance reviews, promotions, and raises in salary, and less tangible incentives such as increased stature or respect.
5	Goals and Feedback	The degree to which goals are clearly communicated, acted upon, and fed back to staff, and alignment of that feedback with goals.
6	Learning Climate	A climate in which: a) leaders express their own fallibility and need for team members' assistance and input; b) team members feel that they are essential, valued, and knowledgeable partners in the change process; c) individuals feel psychologically safe to try new methods; and d) there is sufficient time and space for reflective thinking and evaluation.
E	Readiness for Implementation	Tangible and immediate indicators of organizational commitment to its decision to implement an intervention.
1	Leadership Engagement	Commitment, involvement, and accountability of leaders and managers with the implementation.
2	Available Resources	The level of resources dedicated for implementation and on-going operations, including money, training, education, physical space, and time.
3	Access to Knowledge & Information	Ease of access to digestible information and knowledge about the intervention and how to incorporate it into work tasks.

IV. CHARACTERISTICS OF INDIVIDUALS		
A	Knowledge & Beliefs about the Intervention	Individuals' attitudes toward and value placed on the intervention as well as familiarity with facts, truths, and principles related to the intervention.
B	Self-efficacy	Individual belief in their own capabilities to execute courses of action to achieve implementation goals.
C	Individual Stage of Change	Characterization of the phase an individual is in, as he or she progresses toward skilled, enthusiastic, and sustained use of the intervention.
D	Individual Identification with Organization	A broad construct related to how individuals perceive the organization, and their relationship and degree of commitment with that organization.
E	Other Personal Attributes	A broad construct to include other personal traits such as tolerance of ambiguity, intellectual ability, motivation, values, competence, capacity, and learning style.
V. PROCESS		
A	Planning	The degree to which a scheme or method of behavior and tasks for implementing an intervention are developed in advance, and the quality of those schemes or methods.
B	Engaging	Attracting and involving appropriate individuals in the implementation and use of the intervention through a combined strategy of social marketing, education, role modeling, training, and other similar activities.
4	External Change Agents	Individuals who are affiliated with an outside entity who formally influence or facilitate intervention decisions in a desirable direction.
C	Executing	Carrying out or accomplishing the implementation according to plan.
D	Reflecting & Evaluating	Quantitative and qualitative feedback about the progress and quality of implementation accompanied with regular personal and team debriefing about progress and experience.
EMERGING CONSTRUCTS		
	Faith/Religious Beliefs	Expressions of faith in the context of TB disease, treatment, cessation, or social contexts of the interview participants
	Perceptions of the TB & Tobacco Intervention	Descriptions of what participants remember about the behaviour support and intervention overall, including cytisine, as well as their opinions about it

1. Damschroder LJ, Lowery JC. Evaluation of a large-scale weight management program using the consolidated framework for implementation research (CFIR). Implement. Sci. 2013;8.
